# Improving Gene Knock-In Efficiencies in Sheep Primary Cells Using a CRISPR/Cas9-Gal4 System

**DOI:** 10.3390/cimb47110899

**Published:** 2025-10-29

**Authors:** Yan Li, Sujun Wu, Yunpeng Wu, Yiming Yuan, Yue Zhao, Teng Meng, Wensheng Zhang, Jin Wang, Yefeng Qiu

**Affiliations:** 1 Academy of Military Medical Sciences, Academy of Military Sciences, Beijing 100071, China; scauly@cau.edu.cn (Y.L.); wuyunpeng821@163.com (Y.W.); yuanyimingnice@163.com (Y.Y.); zhaoyuetx@126.com (Y.Z.); mengteng2580@163.com (T.M.); zhangwensheng2024@163.com (W.Z.); 2 College of Animal Science, Shanxi Agricultural University, Taiyuan 030031, China; amywsj@163.com

**Keywords:** CRISPR/Cas9, Gal4, homology-directed repair, sheep fetal fibroblasts

## Abstract

Currently, a major challenge exists in CRISPR-mediated genome editing research in sheep: the low efficiency of exogenous large DNA fragment targeted integration without drug selection or fluorescence enrichment. This restriction significantly impedes the use of precise genome editing in sheep for agricultural, biological, and biomedical purposes. In this study, we employed the strategy of increasing the local concentration of the homologous repair template at the site of the DNA double-strand break (DSB). We achieved this by fusing the DNA binding domain (BD) of the Gal4 protein (Gal4-BD) to the N-terminal end of the SpCas9 protein using a 32-amino acid (aa) flexible linker. Additionally, we incorporated a 17 bp UAS at the 3′ end of the donor template, which can be specifically recognized and bound by Gal-BD. As a result, we observed a significant improvement in the knock-in efficiency of the exogenous large DNA fragment (2997 bp) in sheep fetal fibroblasts (SFFs), increasing it from 5.30% (8/151) to 16.67% (32/192), providing a comparatively efficient and user-friendly method to promote CRISPR-mediated gene knock-in in sheep.

## 1. Introduction

The CRISPR genome editing technology has initiated a significant transformation in biology, medicine, and agriculture due to its ease of use and effectiveness. To date, multiple Cas endonucleases have been developed as versatile tools for precisely manipulating DNA in various organisms. The most commonly used Cas protein is the type II SpCas9 (1368 aa) derived from *Streptococcus pyogenes*, which recognizes the genomic protospacer adjacent motif (PAM) sequence (5′-NGG-3′) guided by CRISPR RNA (crRNA, 20 nt) and trans-activating CRISPR RNA (tracrRNA, 76 nt), or a single guide RNA (sgRNA, 96 nt) formed by the fusion of the two (Figure S1A). It employs two nuclease domains, HNH and RuvC, to cleave the DNA single strands that are complementary and homologous to the crRNA, respectively, resulting in a DSB with blunt ends positioned 3 bp upstream of the PAM [1,2]. The type V-A AsCas12a (1307 aa) from *Acidaminococcus* sp. is another widely used Cas enzyme. Unlike Cas9, Cas12a only needs a single crRNA, which includes a replaceable spacer (23 nt) region and a universal direct repeat (DR, 19 nt) region (Figure S1B). Under the guidance of the crRNA, Cas12a scans the genomic PAM sequence (5′-TTTV-3′), and utilizes its RuvC and Nuc nuclease domains to cleave the target site, generating sticky ends of either 5 or 8 nt, located 14 bp downstream of the PAM. Moreover, in contrast to Cas9, Cas12a has the ability to process its own pre-crRNA into crRNA. Consequently, the primary advantage of the Cas12a system is that it facilitates multiplex genome editing with just one promoter [3,4,5,6,7]. For example, if four different crRNAs targeting distinct genes are arranged in series downstream of the U6 promoter, constructing a single plasmid that co-expresses U6-driven crRNAs and Cas12a enables simultaneous editing of all four target genes. However, the large sizes of Cas9 and Cas12a often restrict the high-efficiency delivery into cells, which impedes AAV-mediated (<4.7 kb) genome editing therapy. The engineered type V-F Cas12f mutant, named CasMINI (529 aa), is compact and less than half the size of Cas9 and Cas12a. Additionally, CasMINI show comparable activities with Cas12a for efficient genome editing. The sgRNA of CasMINI system is also composed of crRNA (23 nt) and tracrRNA (160 nt), where the tracrRNA is fused to the 5′ end of the crRNA, and the structure of its sgRNA is opposite to that of Cas9 system (Figure S1C). The PAM sequence of CasMINI is 5′-TTTR-3′, and the designed targeting site (23 nt) is located at the 3′ end of the PAM, similar to Cas12a [8].

After the CRISPR/Cas enzymes create targeted DSBs in the genome, cells will initiate two major DNA damage repair (DDR) pathways, non-homologous end joining (NHEJ) and homology-directed repair (HDR), in response to the life-threatening lesions. NHEJ is an error-prone repair mechanism that typically results in random base insertions or deletions (InDels) at DSBs. The NHEJ signaling pathway can operate throughout the entire cell cycle without the need for a repair template, making NHEJ-mediated gene knockout highly efficient [9,10]. In many mammalian cells and embryos, the knockout efficiency can reach up to 100% [11,12]. Previously, we employed a Cas9/p53 sgRNA/EGFP co-expression plasmid, along with the enrichment of EGFP^+^ cells through flow cytometry, to achieve up to 100% efficiency in the preparation of genetically mutated sheep fetal fibroblasts cell lines [13]. HDR is an error-free repair mechanism that requires the presence of endogenous homologous chromosomes or exogenous homologous repair templates. This process primarily occurs during the late S and G2/M phases of the cell cycle and competes with NHEJ. In general, cells preferentially repair DSBs through the NHEJ signaling pathway. Consequently, HDR-mediated gene knock-in is typically less efficient than NHEJ-mediated gene knockout and is more significantly influenced by cell type and target location [14,15]. While some researchers have reported knock-in efficiencies exceeding 40% in certain tumor cells, human hematopoietic stem/progenitor cells (HSPCs), pluripotent stem cells (hPSCs), and induced pluripotent stem cells (iPSCs), etc. [10,16,17], the overall gene knock-in efficiency remains quite low for most cell types [2]. This is particularly true for primary somatic cells, such as fibroblasts [13,18,19,20], especially when antibiotic selection or fluorescence enrichment is not employed. Whereas fetal fibroblasts serve as the primary nuclear donor cells in the preparation of genetically modified cloned large animals (cattle, pigs, sheep, and goats) using the somatic cell nuclear transfer (SCNT) technique [21,22], the low HDR efficiency significantly restricts the application of CRISPR-mediated precise genome editing in large animals for agricultural and biomedical purposes.

Increasing the local concentration of double-stranded DNA (dsDNA) or single-stranded oligodeoxynucleotide (ssODN) donor templates at the site of DSBs induced by the CRISPR system is an effective strategy to significantly increase HDR efficiency in cells and embryos. Currently, several methods exist to enrich donor templates at the site of DSBs: (1) the 3′ end of the sgRNA is linked to a 60 nt RNA sequence that is specifically recognized by the Streptavidin/Biotin system, along with the ssODN donor template modified with Streptavidin/Biotin [23]; (2) the C-terminal of Cas9 is fused to an affinity protein (avidin) via a 16 aa flexible linker, with the 5′ end of the ssODN donor template also modified with Biotin [24]; (3) the C-terminal of Cas9 is fused to a SNAP protein, while the ssODN donor template is tagged with O6-benzylguanine (BG), which can specifically bind to the SNAP protein [25]; (4) the C-terminal of SpCas9, CjCas9, and AsCas12a is fused to the DNA binding domain (N57) of the Sleeping Beauty (SB) transposase through a 30 aa linker, respectively, and the dsDNA donor template is linked to a 292 bp of SB-binding sequence [26].

Motivated by this strategy, we aim to develop a more user-friendly donor-template tethering system and apply it to enhance gene knock-in efficiency in SFFs, the most important tool cells for SCNT. Gal4 is a transcriptional activator found in yeast, made up of 881 amino acids. It mainly consists of a DNA binding domain (BD) and activation domains (AD). BD is a peptide consisting of 147 amino acids found at the N-terminal of the Gal4 protein. It is responsible for recognizing and binding specifically to the upstream active sequence (UAS) in the promoter region of Gal4 effector genes, which are related to galactose metabolism, including Gal1, Gal2, and Gal7. The UAS is generally conserved and usually comprises a 17 bp sequence: 5′-CGGRNNRCYNYNYNCNCCG-3′ [27,28,29]. LexA functions as a vital repressor protein within the SOS response mechanism of *Escherichia coli*, consisting of 202 amino acids. LexA is structured with an N-terminal DNA binding domain and a C-terminal catalytic core domain. The BD of LexA specifically engages with the SOS box, also known as the operator (op), which is characteristically a conserved 16 bp palindromic sequence (5′-CTGTN_8_ACAG-3′) [28,30,31]. In this study, to improve the HDR efficiency in SFFs, we commenced by evaluating the genome editing capabilities of three distinct Cas endonucleases: SpCas9, AsCas12a, and CasMINI, from which we identified the most effective enzyme. Following this, we fused the DNA binding domains of the Gal4 and LexA proteins to the chosen enzyme, respectively. Our findings revealed that the fusion of the Gal4-BD to the N-terminal of Cas9, linked by a 32 aa flexible linker, significantly increased the CRISPR-mediated gene knock-in efficiency by 3.15-fold (from 5.30% (8/151) to 16.67% (32/192), *p* = 3.63 × 10^−3^) in SFFs when the 3′ end of the homologous repair template included the UAS, thereby providing a comparatively efficient and user-friendly strategy for gene knock-in in SFFs.

## 2. Materials and Methods

### 2.1. Plasmid Construction

To construct the CasMINI-Puro plasmid (sgRNA, CasMINI, and puromycin co-expression), the U6/tracrRNA and CasMINI gene sequences were initially synthesized by Tsingke Biotech (Beijing, China). Following this, the U6/tracrRNA fragment was ligated into the commercial pEGFP-N1 (Clontech, Beijing, China) backbone plasmid after undergoing AseI single enzyme digestion through seamless cloning, resulting in the formation of the pEGFP-N1-U6/tracrRNA plasmid. Finally, the CasMINI fragment, in conjunction with the T2A-Puro fragment amplified from the commercial pX459 plasmid (Addgene plasmid 48139, Watertown, MA, USA; sgRNA, Cas9, and puromycin co-expression), were ligated into the pEGFP-N1-U6/tracrRNA backbone plasmid, which had been subjected to dual enzyme digestion (EcoRI and NotI), utilizing the seamless cloning technique.

To construct the pX330-LexA/Cas9 and pX330-Gal4/Cas9 plasmids, the gene sequences for LexA and Gal4 were designed to include a SV40 nuclear localization signal (NLS) at the 5′ end, followed by the LexA/Gal4 DNA binding domain, two linked BsaI cleavage site sequences at the 3′ end, separated by four bases of AGTC, and synthesized by Tsingke Biotech. Subsequently, the LexA and Gal4 fragments were ligated into the pX330 backbone plasmid (Addgene plasmid 42230; sgRNA and Cas9 co-expression), which had been cleaved by AgeI and BglII enzymes, utilizing the seamless cloning approach, respectively.

To construct the pX330-Cas9/LexA and pX330-Cas9/Gal4 plasmids, the gene sequences for LexA and Gal4 were initially synthesized by Tsingke Biotech. These sequences included two contiguous BsaI cleavage site sequences, separated by four bases of AGTC at the 5′ end, followed by the DNA binding domains of LexA/Gal4. Following gene synthesis, the LexA and Gal4 fragments were ligated into the pX330 backbone plasmid after undergoing double cleavage with BsmI and EcoRI enzymes, respectively, using the seamless cloning technique.

To construct the pX330-LexA/linker/Cas9, pX330-Cas9/linker/LexA, pX330-Gal4/linker/Cas9, and pX330-Cas9/linker/Gal4 plasmids, a total of 20 pairs of forward and reverse primers, which included the five distinct linkers, were synthesized by Tsingke Biotech. Following this, the 20 pairs of primers were subjected to phosphorylation and subsequent annealing. The resulting 20 annealed products were then ligated using T4 DNA ligase into the respective plasmids that had been cleaved with BsaI: pX330-LexA/Cas9, pX330-Cas9/LexA, pX330-Gal4/Cas9, and pX330-Cas9/Gal4.

To construct the 36 gene knockout plasmids, two Cas9/sgRNAs, two Cas12a/crRNAs, and two CasMINI/sgRNAs were designed to target the exons of the sheep MSTN gene (NCBI Gene ID: 443449) and FGF5 gene (NCBI Gene ID: 100642180), respectively, using the CRISPOR program (https://crispor.gi.ucsc.edu, accessed on 24 October 2025). Following the synthesis of the sgRNA/crRNA oligos, the phosphorylation and annealing of 12 pairs of primers were conducted. These primers were then ligated into the respective plasmids that had been cleaved with a single enzyme: pX459 (BbsI), pY30 (BsmBI, Addgene plasmid 84745; crRNA, Cas12a, and puromycin co-expression), and CasMINI-Puro (BsmBI), utilizing T4 DNA Ligase. Additionally, the annealed oligo products of Cas9/MSTN sgRNA2 and Cas9/FGF5 sgRNA2 were ligated into the BbsI single enzyme cleaved pX330 and pX330-Gal4/linker/Cas9 plasmids, respectively. Similarly, the previously designed Cas9/MSTN sgRNA [13] was also ligated into the BbsI single enzyme cleaved pX330-LexA/linker/Cas9, pX330-Cas9/linker/LexA, pX330-Gal4/linker/Cas9, and pX330-Cas9/linker/Gal4 plasmids, respectively. The detailed oligo sequences are listed in Table S1.

To construct the FGF5/SV40 LT donor plasmid, the commercial pBABE-puro SV40 LT plasmid (Addgene plasmid 13970; Containing the SV40 LT gene expression construct) was initially subjected to PCR amplification (SV40 LT gene). Then, the mCherry gene in the commercial pmCherry-N1 (Clontech) plasmid was replaced with the SV40 LT gene using the seamless cloning technique, thereby resulting in the formation of the pSV40 LT-N1 plasmid. Subsequently, PCR amplification of the pSV40 LT-N1 plasmid was performed to obtain the CMV-SV40 LT-pA gene expression structure. Additionally, PCR amplification of the sheep genome was performed to acquire the left and right homology arm sequences of the FGF5/sgRNA2 tagert, each measuring 2 kb in length. Finally, the left and right homology arms, along with the CMV-SV40 LT-pA fragment were subcloned into the pUC57 backbone plasmid using seamless cloning technology. Similarly, to construct the MSTN/mCherry donor plasmid, the pmCherry-N1 plasmid underwent PCR amplification to produce the CMV-mCherry-pA gene expression structure. Simultaneously, the sheep genome was PCR amplified to obtain the left and right homology arm sequences, each measuring 1 kb in length, at the MSTN/sgRNA2 target. Following this, seamless cloning technique was employed to subclone the left and right homology arms, in conjunction with the CMV-mCherry-pA fragment, into the pUC57 backbone plasmid.

The descriptions and applications of the plasmids are listed in Table S2.

### 2.2. Cell Culture and Transfection

SFFs were derived from a 30-day-old Dorper sheep fetus and cultured in DMEM/F12 (Dulbecco’s Modified Eagle Medium: Nutrient Mixture F-12; Gibco, Waltham, MA, USA) supplemented with 10% FBS (fetal bovine serum; Gibco) and 1% penicillin/streptomycin (Gibco) at 37 °C with 5% CO_2_. For the derivation, scissors were first used to finely chop the sheep fetal skin tissue and evenly distribute the tissue fragments to ensure they adhere to the bottom of the culture dish. Next, DMEM/F12 medium supplemented with 10% FBS and 1% penicillin/streptomycin was added for cultivation (Figure S2A). Finally, once the cells in the dish reached confluence, the SFFs were isolated and purified using the differential adhesion method. Primary fibroblasts isolated from the fetus constitute the first-generation cells. Subsequent passages are performed at a 1:4 split ratio, with each passage taking approximately three days.

All nucleofection experiments were performed by the Lonza Nucleofector 2b Device with Amaxa^TM^ Basic Nucleofector^TM^ Kit for Primary Fibroblasts (Lonza, Guangzhou, China), following the manufacturer’s instructions. In brief, the mixture of SFFs (1.5 × 10^6^) and DNA (plasmid, donor template) was resuspended in 100 μL of NucleofectorTM Solution and transfected using program A-033. For the MSTN and FGF5 gene knockout experiments, 15 μg of individual plasmid (pX459-MSTN/sgRNA1, pX459-MSTN/sgRNA2, pX459-FGF5/sgRNA1, pX459-FGF5/sgRNA2; pY30-MSTN/sgRNA1, pY30-MSTN/sgRNA2, pY30-FGF5/sgRNA1, pY30-FGF5/sgRNA2; CasMINI-Puro-MSTN/sgRNA1, CasMINI-Puro-MSTN/sgRNA2, CasMINI-Puro-FGF5/sgRNA1, CasMINI-Puro-FGF5/sgRNA2) was used to transfect the tenth-generation SFFs. For the T2A-EGFP gene knock-in experiments, 2.5 pmol of MSTN gene knockout plasmid (pX330-MSTN/sgRNA, pX330-LexA/linker/Cas9-MSTN/sgRNA, pX330-Cas9/linker/LexA-MSTN/sgRNA, pX330-Gal4/linker/Cas9-MSTN/sgRNA, pX330-Cas9/linker/Gal4-MSTN/sgRNA) and 6 pmol of repair template (MSTN-T2A-EGFP with 1000 bp HA, MSTN-T2A-EGFP with 1000 bp HA containing the 16 bp op sequence at either the 5′ or 3′ end, MSTN-T2A-EGFP with 1000 bp HA containing the 17 bp UAS at either the 5′ or 3′ end) amplified from the donor plasmid were used to transfect the fifth-generation SFFs. For the mCherry gene knock-in experiment, 2.5 pmol of MSTN gene knockout plasmid (pX330-MSTN/sgRNA2, pX330-Gal4/linker/Cas9-MSTN/sgRNA2) and 6 pmol of repair template (CMV-mCherry-pA with 1000 bp HA, CMV-mCherry-pA with 1000 bp HA containing the 17 bp UAS sequence at the 3′ end) amplified from the donor plasmid were used to transfect the second-generation SFFs. For the SV40 LT gene knock-in experiment, 2.5 pmol of FGF5 gene knockout plasmid (pX330-FGF5/sgRNA2, pX330-Gal4/linker/Cas9-FGF5/sgRNA2) and 6 pmol of repair template (CMV-SV40 LT-pA with 2000 bp HA, CMV-SV40 LT-pA with 2000 bp HA containing the 17 bp UAS at the 3′ end) amplified from the donor plasmid were used to transfect the second-generation SFFs. Following nucleofection, the SFFs were transferred to 6-well plates for subsequent culture and analysis. The detailed PCR primer sequences used for generation of the donor templates are listed in Table S1.

### 2.3. Determination of the Minimum Lethal Dose of Puromycin in SFFs

The tenth-generation SFFs were inoculated into a 96-well cell culture plate, ensuring an equal number of cells per well. Upon reaching 60% confluence, the SFFs were divided into 11 groups. One group continued to be cultured without any treatment, while the remaining 10 groups were transitioned to a culture medium containing varying concentrations of puromycin: 100 ng/mL, 200 ng/mL, 300 ng/mL, 400 ng/mL, 500 ng/mL, 600 ng/mL, 700 ng/mL, 800 ng/mL, 900 ng/mL, and 1000 ng/mL. After 48 h of administration, when all the cells in one of the experimental groups had undergone shedding and subsequent death, cell viability in each treatment group was assessed by MTT assay using the Cell Proliferation and Cytotoxicity Assay Kit (Beyotime, Shanghai, China) in accordance with the manufacturer’s instructions.

### 2.4. Determination of the Gene Knockout Efficiency

For the MSTN and FGF5 gene knockout assays, genomic DNA was extracted from each group transfected with the pX459/pY30/CasMINI-Puro plasmid and selected with puromycin, using the Takara (Shanghai, China) MiniBEST Universal Genomic DNA Extraction Kit following the manufacturer’s protocol. Specific forward and reverse primers were designed around the targeted sites. PCR amplification was then performed through Takara PrimeSTAR DNA Polymerase, and the resultant PCR products containing mutations were directly subcloned into the pTOPO-Blunt Cloning Vector (Aidlab, Beijing, China). Forty individual bacterial colonies from each sample were randomly selected and sent to Tsingke Biotech for Sanger sequencing. The sequencing results were subsequently compared to the wild-type sequences to assess the mutation efficiencies [13,32], sequencing failures were excluded from the analysis. The detailed PCR primer sequences used for detection are listed in Table S1.

### 2.5. Determination of the Gene Knock-In Efficiency

For the T2A-EGFP gene knock-in assays, after 48 h of culture in 6-well plates, the transfected cells were harvested through trypsin digestion and subsequently resuspended in 300 µL of PBS with 1% FBS, to which 2.5 µL of 7-AAD (BD Biosciences, Shanghai, China) was added. Data were collected utilizing a BD FACSVerse flow cytometer. Live cells were identified through gating based on forward scatter area and side scatter area, while single cells were distinguished by employing forward scatter width in relation to forward scatter area. The quantification of EGFP-positive cells was achieved by gating the relevant channel, using EGFP-negative cells as a control. All FACS data were analyzed using FlowJo software (version 11) (BD Biosciences).

For the mCherry and SV40 LT gene knock-in assays, after 48 h of transfection, cells cultured in 6-well plates were collected and single live cells were sorted into 96-well plates using a BD FACSAria III flow cytometer and cultured in DMEM/F12 supplemented with 20% FBS and 1% penicillin/streptomycin/amphotericin (Gibco) (Figure S2B). Following 14 d of cell culture, each cell colony was partially lysed using a lysis buffer (4% Tris-HCl (1 M, PH = 8.0), 0.9% NP-40, 0.9% Triton X-100, and 0.4 mg/mL Proteinase K; 65 °C for a duration of 30 min, followed by an increase to 95 °C for 15 min, and subsequently held at 4 °C), and PCR genotyping was conducted to identify the targeted integration SFFs lines using primer #1 (mCherry) and primer #2 + #3 (SV40 LT), respectively (Table S1).

### 2.6. Fluorescence Microscopy and Immunofluorescence

The mCherry and SV40 LT knock-in SFFs were cultured in 8-well glass chamber slides (Millipore, Billerica, MA, USA), with wild type SFFs serving as a control. Upon reaching approximately 70% confluence, the samples were fixed using 4% paraformaldehyde at room temperature (RT) for 15 min. The mCherry knock-in cells were subsequently stained with DAPI (Beyotime) for 2 min at RT and imaged directly using the EVOS^TM^ M5000 Imaging System (Invitroge, Waltham, MA, USA). In contrast, the SV40 LT knock-in cells underwent a series of labeling procedures, which included permeabilization with 0.1% Triton X-100, incubation with a primary antibody (Anti-SV40 LT-antigen–antibody; Abcam, 234426, Cambridge, UK) at a dilution of 1:50 for 2 h at RT, followed by incubation with an AF488-labeled goat anti-rabbit secondary antibody (Beyotime, A0423) at a dilution of 1:1000 for 1 h at RT, and subsequent DAPI staining. The expression of SV40 LT was imaged using a Nikon A1 confocal microscope (Nikon, Tokyo, Japan). The mean fluorescence intensity in images of mCherry and SV40 LT gene knock-in cell lines was measured using ImageJ software (version 1.54) [33].

### 2.7. Off-Target Analyses

For the sheep MSTN/sgRNA2 and FGF5/sgRNA2 designed and employed, the Cas-OFFinder algorithm (http://www.rgenome.net/cas-offinder/, accessed on 24 October 2025) was used to identify and select the five potential off-target sites with the highest similarity in the sheep genome. For each potential off-target site, two sets of primers were designed to perform PCR testing on SFFs positive for the mCherry or SV40 LT gene knock-in. One set of primers flanked each potential off-target site; after PCR amplification, Sanger sequencing was conducted to detect any base mutations. The other set included forward primers positioned upstream of each potential off-target site and reverse primers located within the inserted mCherry or SV40 LT gene. Following PCR amplification, the presence or absence of PCR bands was assessed to determine whether any imprecise insertion of the mCherry or SV40 LT gene had occurred.

### 2.8. Statistical Analysis

For the T2A-EGFP gene knock-in and MTT assays, values are presented as mean ± SD. The percentage data were first transformed using the arcsine square root transformation (arcsin$\sqrt{p}$). Subsequently, assumptions of normality and homoscedasticity were evaluated. If these assumptions were satisfied, a one-way ANOVA was conducted to assess overall significance, followed by Dunnett’s test for multiple comparisons against the control group (Cas9/LexA, LexA/Cas9, and Gal4/Cas9). When variances were unequal, Welch’s ANOVA was applied instead (Cas9/Gal4, MTT) (SPSS, version 30).

For the mCherry and SV40 LT gene knock-in assays, the knock-in efficiency data were analyzed using χ^2^-test for comparisons between two groups (SPSS).

Differences were deemed statistically significant at * *p* < 0.05 and ** *p* < 0.01.

## 3. Results

### 3.1. Determining and Comparing the Cleavage Efficiencies of SpCas9, AsCas12a, and CasMINI at the MSTN and FGF5 Loci in SFFs

**Figure 1:**
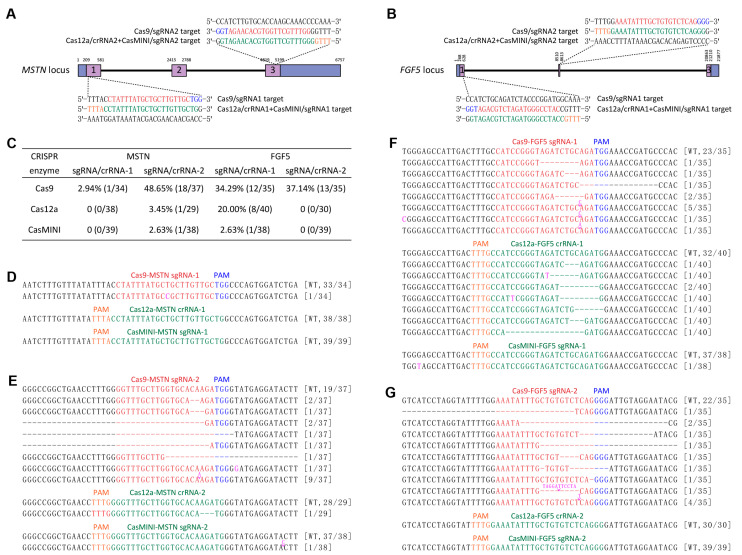
Determining and comparing the cleavage efficiencies of SpCas9, AsCas12a, and CasMINI at the *MSTN* and *FGF5* loci in SFFs. (**A**,**B**) Schematic of SpCas9/crRNAs and AsCas12a+CasMINI/crRNAs specific to exon 1 and 3 of the *MSTN* locus and exon 1 and 2 of the *FGF5* locus. The SpCas9/crRNAs and AsCas12a+CasMINI/crRNAs sequences are indicated in red and green typeface, and the corresponding PAM in blue and orange, respectively. (**C**) Summary of gene knockout efficiencies of the SpCas9, AsCas12a, and CasMINI at the *MSTN* and *FGF5* loci in SFFs. (**D**–**G**) TA cloning and Sanger sequencing analysis of the mutation efficiencies and types detected in the pX459-MSTN/FGF5, pY30-MSTN/FGF5, or CasMINI-puro-MSTN/FGF5 gene knockout plasmids electroporated and puromycin selected SFFs. The newly added nucleotides are highlighted in pink, and deletions are indicated by a dashed line (-). The colony numbers are surrounded by brackets.

In this study, firstly we conducted an assessment of the targeting efficiencies of SpCas9, AsCas12a, and CasMINI in SFFs. We selected MSTN and FGF5 as target genes due to their significant agricultural value and frequent use in sheep genome editing research. MSTN, part of the transforming growth factor-β (TGF-β) superfamily, serves as a negative regulator of muscle development. Both natural and artificially induced mutations in MSTN have been found to boost muscle mass by about 20% in various animals. FGF5, a member of the fibroblast growth factor (FGF) superfamily, functions as a negative regulator of hair growth. Naturally and manually occurring mutations in FGF5 in many species have been shown to significantly increase hair length [13,34]. We designed two target sequences for each of the sheep MSTN and FGF5 genes. For each target, the crRNA sequences for AsCas12a and CasMINI were identical and encompassed the crRNA sequences for SpCas9. Specifically, the target sites within the MSTN gene were located in exons 1 and 3, while the target sites within the FGF5 gene were situated in exons 1 and 2 (Figure 1A,B). Following this, the designed crRNAs were cloned into their respective plasmids: pX459 for SpCas9, pY30 for AsCas12a, and CasMINI-Puro for CasMINI (Figure S1A–C). Furthermore, we determined the minimum lethal dose of puromycin in SFFs; microscopic observations and MTT assays revealed that the minimum lethal dose of puromycin for the treated SFFs was 900 ng/mL, with a treatment duration of 48 h (Figure S3A,B).

Subsequently, we nucleofected the three kinds of gene knockout plasmids (pX459-MSTN/FGF5, pY30-MSTN/FGF5, and CasMINI-Puro-MSTN/FGF5) into SFFs, respectively. Following puromycin selection (900 ng/mL, 48 h), TA cloning and Sanger sequencing assays revealed that SpCas9 exhibited the highest knockout efficiency in SFFs, with mutation rates for the four target sites within the MSTN and FGF5 genes recorded at 2.94% (1/34), 48.65% (18/37), 34.29% (12/35), and 37.14% (13/35), respectively. In contrast, AsCas12a and the novel genome editing enzyme CasMINI were only capable of cleaving DNA double strands at the second target site of the MSTN gene (AsCas12a: 3.45%, 1/29; CasMINI: 2.63%, 1/38) and the first target site of the FGF5 gene (AsCas12a: 20.00%, 8/40; CasMINI: 2.63%, 1/38), and the mutation rates at the remaining two sites were observed to be zero (Figure 1C–G). Consequently, the classical gene editing enzyme SpCas9 was selected for the subsequent experiments in this study.

### 3.2. Effect of Fusing LexA-BD Peptide to the C- or N-Terminal of Cas9 Protein on the Efficiency of Gene Knock-In in SFFs

**Figure 2:**
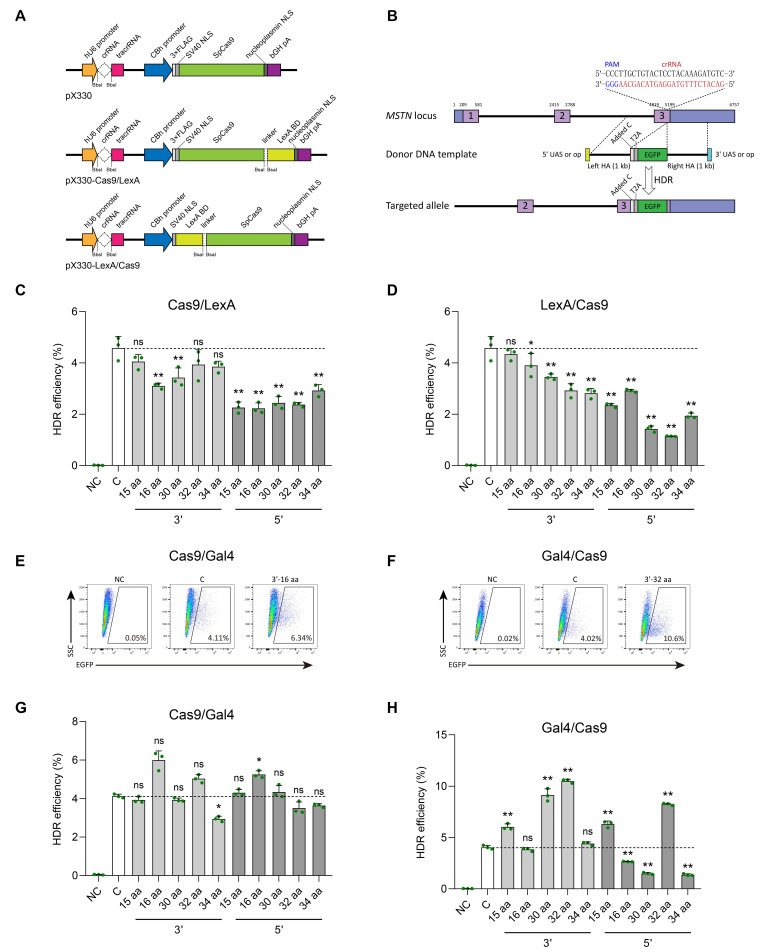
Effect of fusing LexA-BD or Gal4-BD peptide to the C- or N-terminal of Cas9 protein on the efficiency of gene knock-in in SFFs. (**A**) Schematic structures of the pX330, pX330-Cas9/LexA, and pX330-LexA/Cas9 plasmids. LexA-BD peptide was fused to the C- or N-terminal of the Cas9 protein by five different flexible linkers. (**B**) Schematic of the targeting strategy for knock-in T2A-EGFP with 1000 bp HA repair template containing the op or UAS at either the 5′ or 3′ end to the sheep *MSTN* locus. (**C**,**D**) FACS analysis of EGFP-positive cells at 48 h after co-transfection of T2A-EGFP with 1000 bp HA donor template and pX330 gene knockout plasmid (control group, (**C**)), T2A-EGFP with 1000 bp HA donor template containing the op sequence at either the 5′ or 3′ end and pX330-Cas9/LexA (C-terminal fusion groups) or pX330-LexA/Cas9 (N-terminal fusion groups) gene knockout plasmid into SFFs, respectively. The T2A-EGFP with 1000 bp HA donor template transfected cells were used as a negative control (NC). (**E**–**H**) FACS analysis of EGFP-positive cells at 48 h after co-transfection of T2A-EGFP with 1000 bp HA donor template and pX330 gene knockout plasmid, T2A-EGFP with 1000 bp HA donor template containing the UAS at either the 5′ or 3′ end and pX330-Cas9/Gal4 (C-terminal fusion groups) or pX330-Gal4/Cas9 (N-terminal fusion groups) gene knockout plasmid into SFFs, respectively. Representative FACS results are presented. *n* = 3 biological replicates. Error bars represent SD. ns indicates no significant difference, * indicates *p* < 0.05, ** indicates *p* < 0.01.

We next investigated whether the fusion of LexA-BD or Gal4-BD peptide with Cas9 protein could improve the HDR efficiency without drug selection or fluorescence enrichment. We initially fused LexA-BD to either the C-terminal or N-terminal of Cas9 protein (Figure 2A), utilizing each of the five commonly employed flexible linkers in genome editing research (GGS×5, 15 aa; XTEN, 16 aa [35]; SA-GGGGS×3-G-NLS^SV40^-AAAGS, 30 aa [36]; SGGS×2-XTEN-SGGS×2, 32 aa [37,38,39]; SGGS×2-NLS^SV40^-SGGS×2, 34 aa [40]). To guarantee the precision of the knock-in efficiency evaluating assay, we utilized a CRISPR/T2A-EGFP fluorescent reporter knock-in system developed previously in SFFs [13]. The successful integration of the T2A-EGFP with 1000 bp homology arm (HA) repair template into exon 3 of the sheep MSTN gene, facilitated by Cas9/MSTN sgRNA/HDR, will result in the action of the T2A self-cleaving peptide. This peptide will enable the hydrolysis of a full-length mRNA, transcribed from the endogenous promoter of the MSTN gene, into two distinct proteins upon translation: the MSTN mutant protein and the fully functional EGFP green fluorescent protein (Figure 2B). Therefore, the efficiency of gene knock-in will be determined through flow cytometry analysis.

In brief, based on the highly efficient transfection strategy (95.07% ± 0.29%) we previously established in SFFs [13], we performed nucleofection of the pX330-MSTN/sgRNA plasmid and T2A-EGFP with 1000 bp HA repair template into SFFs, which served as control group. Additionally, the five distinct linker-linked pX330-Cas9/LexA-MSTN/sgRNA or pX330-LexA/Cas9-MSTN/sgRNA plasmids, along with the corresponding T2A-EGFP with 1000 bp HA repair template containing a 16 bp op sequence (5′-CTGTATGATCATACAG-3′) at either the 5′ or 3′ end, were also subjected to nucleofection in SFFs, respectively. After 48 h of co-transfection, FACS results showed that none of the 20 fusion groups exhibited a higher rate of green fluorescence compared to the control group (4.58% ± 0.45%, *n* = 3) (Figure 2C,D and Figure S4C,D; Table S3). Notably, the combination of the LexA protein with the Cas9 protein does not improve the gene knock-in efficiency; instead, it may modify the conformation of the Cas9 protein, consequently diminishing its genome editing efficacy.

### 3.3. Effect of Fusing Gal4-BD Peptide to the C- or N-Terminal of Cas9 Protein on the Efficiency of Gene Knock-In in SFFs

We further fused Gal4-BD to either the C-terminal or N-terminal of Cas9 protein by employing the five flexible linkers mentioned above (Figure S4A,B). Similar to LexA, we also co-transfected the T2A-EGFP with 1000 bp HA donor template and pX330-MSTN/sgRNA gene knockout plasmid (control group, C), T2A-EGFP with 1000 bp HA donor template containing the UAS (5′-CGGAAAGCTTCCTTCCG-3′) at either the 5′ or 3′ end and pX330-Cas9/Gal4-MSTN/sgRNA (C-terminal fusion groups) or pX330-Gal4/Cas9-MSTN/sgRNA (N-terminal fusion groups) gene knockout plasmid into SFFs, respectively. At 48 h post co-transfection, FACS analysis revealed that the proportion of EGFP-positive cells, gated from live cells (7-AAD negative), demonstrated that the combination of Gal4 with Cas9 resulted in a notable enhancement of gene knock-in efficiency (Figure 2E–H, Figures S4E,F and S5; Table S3). In particular, the fusion of the Gal4-BD to the N-terminal of the Cas9 protein through the 32 amino acid linker peptide (SGGS×2-XTEN-SGGS×2), along with the modification of the 3′ end of the donor template to incorporate the UAS, led to a substantial increase in knock-in efficiency from 4.05% ± 0.19% to 10.50% ± 0.17% (*n* = 3, *p* = 2.10 × 10^−9^), representing an approximate 2.60-fold improvement relative to the control group. It is worth noting that, although other untested flexible linkers may yield better results, in this study we selected this 32 amino acid linker for subsequent experiments and named this efficient gene knock-in system as CRISPR/Cas9-Gal4.

### 3.4. CRISPR/Cas9-Gal4 System Promote the Generation of Fluorescent Protein Gene Targeted Integration SFFs Lines

**Figure 3:**
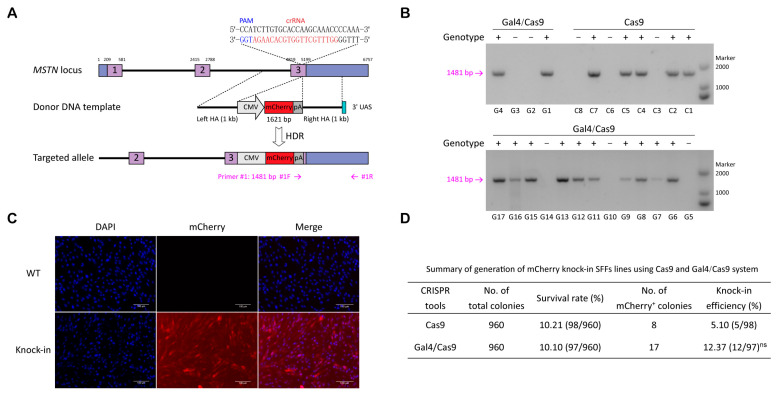
CRISPR/Cas9-Gal4 system promote the generation of fluorescent protein gene targeted integration SFFs lines. (**A**) Schematic of the targeted integration strategy for knock-in exogenous *mCherry* gene to the sheep *MSTN* locus. The fragment for integration measures 1621 bp in length, the left and right homology arms each measure 1000 bp in length, and the 3′ end of the right homology arm contains the 17 bp UAS. The PCR detection forward primer #1F was designed in the SV40 poly(A) signal, while the reverse primer #1R was designed outside of the flanking homology arm. (**B**) PCR genotyping results of the experimental and control groups. The positive monoclonal cell lines were first screened under the fluorescence microscopy, followed by lysis of a portion of cells and further detected by PCR. +, *mCherry* knock-in; −, negative. C1 to C8 represent eight single-cell colonies in the control group, while G1 to G17 represent seventeen single-cell colonies in the Gal4/Cas9 group. (**C**) Red fluorescence expression in *mCherry* gene knock-in (fifth-generation) and wild type SFFs. mCherry, red; DNA, blue. (**D**) Summary of the preparation of *mCherry* gene knock-in SFFs lines utilizing the CRISPR/Cas9 and CRISPR/Cas9-Gal4 systems. The knock-in efficiency data were analyzed using χ^2^ test for comparisons between two groups, revealing no significant difference between them (ns, *p* = 0.13).

Having demonstrated the superior performance of the CRISPR/Cas9-Gal4 system in HDR, we applied this system to generate monoclonal cell lines of SFFs for the purpose of assessing its practicability (Figure 3A). Based on the identified efficiently mutated target site (sgRNA2, 48.65%) in the MSTN gene, we constructed CMV-mCherry-pA HDR donor plasmid that contain 1 kb of left and right homology arms. The pX330-MSTN/sgRNA2 plasmid and the CMV-mCherry-pA with 1000 bp HA repair template co-transfected SFFs were designated as the control group, and the pX330-Gal4/32 aa/Cas9-MSTN/sgRNA2 plasmid and the CMV-mCherry-pA with 1000 bp HA repair template containing the UAS at the 3′ end co-transfected SFFs were designated as the experimental group. At 48 h post-transfection, we utilized flow cytometry to sort single live cells of the two groups into 96-well plates, respectively. After 14 days of cell culture, the observation of mCherry^+^ single-cell colonies by fluorescence microscope and PCR genotyping results showed that there was no obvious difference in the rate of monoclonal formation between the experimental and control groups. While the knock-in efficiency of mCherry gene did not reach statistical significance (*p* = 0.13), a notable enhancement was evident, with efficiency increasing by 2.43-fold from 5.10% (5/98) to 12.37% (12/97) (Figure 3B,D; Table S3). Subsequently, we selected a highly active positive cell line from the Gal4/Cas9 group for further validation. Sequencing result confirmed the correct integration of CMV-mCherry-pA at the MSTN locus in SFFs (Figure S6A). Fluorescence microscopy results showed that the expression of mCherry protein remained robust in both the fifth-generation and twentieth-generation positive SFFs (Figure 3C and Figure S6B), the mean fluorescence intensities were 49.05 AU and 39.77 AU, respectively (Figure S6C). Off-target analyses indicated that no mutations or gene knock-in events occurred at any of the five potential off-target sites in the fifth-generation positive SFFs (Figure S6D).

### 3.5. CRISPR/Cas9-Gal4 System Promote the Generation of Exogenous Large DNA Fragment Targeted Integration SFFs Lines

**Figure 4:**
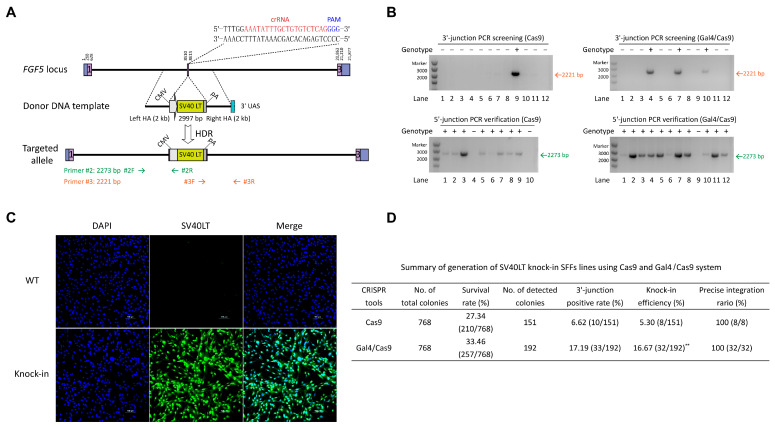
CRISPR/Cas9-Gal4 system promotes the generation of exogenous large DNA fragment targeted integration SFFs lines. (**A**) Schematic of the targeting strategy for knock-in CMV-SV40LT-pA with 2000 bp HA (3′ end of the right HA contains the 17 bp UAS) to the sheep *FGF5* locus. Primers #2R and #3F were internal reverse and forward primers in the CMV promoter and SV40 poly(A) signal, respectively, while primers #2F and #3R were both designed outside of the flanking homology arms. (**B**) Representative PCR genotyping results of the experimental and control groups. +, *SV40 LT* knock-in; −, negative. For each group of samples, PCR amplification at the 3′-junction was initially performed to screen for positive samples, followed by additional PCR validation at the 5′-junction. Only samples exhibiting double positive PCR results at both the 3′- and 5′-junctions were identified as gene knock-in monoclonal cell lines. (**C**) Detection of the *SV40LT* gene expression by IF in 3′- and 5′-junctions positive SFFs (fifth-generation). The wild type SFFs were used as a negative control. SV40 LT, green; DNA, blue. Scale bar = 100 μm. (**D**) Summary of generation of *SV40 LT* knock-in SFFs lines using Cas9 and Gal4/Cas9 system. Only those with correct Sanger sequencing results for the 5′ end PCR amplification products were confirmed to have precise SV40 LT integration. The knock-in efficiency data were analyzed using χ^2^ test for comparisons between two groups, revealing a highly significant difference between them (**, *p* = 3.63 × 10^−3^).

Due to the heterogeneity of each locus, we repeatedly selected another target site (FGF5/sgRNA2, 37.14%) to further validate the practicality of the CRISPR/Cas9-Gal4 system in enhancing the efficiency of exogenous large DNA fragment knock-in (Figure 4A). Similar to the MSTN locus, we first constructed a SV40 LT gene expression HDR donor plasmid (CMV-SV40 LT-pA that contain 2 kb of left and right homology arms). In a comparable manner, we established both a control group (SFFs co-transfected with pX330-FGF5/sgRNA2 plasmid and CMV-SV40 LT-pA with 2000 bp HA repair template) and an experimental group (pX330-Gal4/32 aa/Cas9-FGF5/sgRNA2 plasmid and CMV-SV40 LT-pA with 2000 bp HA repair template containing the UAS at the 3′ end). Following the process of flow sorting single live cells into 96-well plates and a subsequent 14 days of cell culture, a portion of each monoclonal cell line was lysed and then identified the occurrence of correct integration by PCR. PCR genotyping results indicated that the knock-in efficiency of the large fragment SV40 LT (2997 bp) in the experimental group reached up to 16.67% (32/192), representing a 3.15-fold increase compared to the control group (5.30% (8/151), *p* = 3.63 × 10^−3^) (Figure 4B,D; Table S3). Further Sanger sequencing of the 5′-end PCR amplification products revealed that SV40 LT was precisely integrated into the FGF5 locus in eight positive monoclonal cell lines from the Cas9 group, as well as in thirty-two positive monoclonal cell lines from the Gal4/Cas9 group (Figure 4D and Figure S7A). Afterwards, we chose a highly active positive cell line from the Gal4/Cas9 group for further validation. Sanger sequencing of the 3′-end PCR amplification product and immunofluorescence (IF) provided additional confirmation of the precise insertion of SV40 LT gene (Figure 4C and Figure S7A), the mean fluorescence intensity of the SV40 LT expression was 193.94 AU, while the control group showed 3.47 AU (Figure S7B). Off-target analyses indicated that no mutations or gene knock-in events occurred at any of the five potential off-target sites in the SV40 LT gene knock-in SFFs (Figure S7C). Taken together, these results suggest that CRISPR/Cas9-Gal4 system has a prominent application in the efficient gene knock-in in sheep.

## 4. Discussion

The CRISPR system-mediated precise genome editing, which operates through the homology-directed repair pathway, holds significant potential for the improvement of important economic traits and the enhancement of disease resistance in large animals, including pigs, cattle, sheep, and goats. This innovative technology also can facilitate the generation of large animal bioreactors and large animal models of human disease [22,41,42]. However, in the primary somatic cells of large animals, such as fibroblasts, the HDR efficiency of conventional strategies is notably low in the absence of drug selection or fluorescence enrichment. This phenomenon can be attributed to the predominant occurrence of NHEJ repair, which competes with HDR as an alternative pathway for the repair of DSBs. This constraint poses a considerable obstacle to the efficient generation of precise genetically modified large animals utilizing the CRISPR genome editing system in combination with pronuclear microinjection or somatic cell nuclear transfer approaches. In studies involving CRISPR system-mediated precise genome editing, a high frequency of DSBs at the interest site is crucial for efficient exogenous gene knock-in [43]. Therefore, in this study, we commenced by evaluating the gene knockout capabilities of different CRISPR systems in order to identify the Cas nuclease that can proficiently induce DNA DSBs in sheep primary cells. Following over ten years of development and optimization, three primary CRISPR families have been recognized for their ability to induce site-specific genomic DSBs: Cas9 (SpCas9 [2], SaCas9 [44], StCas9 [45], CjCas9 [46], NmCas9 [47], etc.), Cas12 (AsCas12a [48], LbCas12a [49], Cas12d/CasY [50], Cas12e/CasX [51], Cas12j/CasΦ [52], etc.), and engineered Cas14 (CasMINI [8]). We selected a representative and commonly employed Cas nuclease (SpCas9, AsCas12a, CasMINI) from each family to perform mutation efficiency detection assays at four loci within two genes, and found that the classical SpCas9 protein exhibited the highest efficacy in cleaving dsDNA at the cellular level in sheep.

Multiple strategies can enhance the efficiency of CRISPR/HDR-mediated gene knock-in. For instance, at the DNA level, researchers have optimized the design of homology repair templates by employing linearized dsDNA donor templates devoid of redundant sequences [53], incorporating crRNAs that are identical to the target site at both the 5′ and 3′ ends of the donor templates [54], introducing C6-PEG10 modifications at the 5′ end of the donor templates [55], and opting for ssODN as donor templates in place of dsDNA [56], and optimized the length of the homology arms and the amount of donor templates [57,58], which have been shown to significantly improve the HDR efficiency. At the RNA level, the gene silencing of several key factors in the NHEJ signaling pathway, including Ku70, Ku80, XRCC4, and Ligase IV, through RNAi technology, can markedly improve the integration efficiency of exogenous genes by 1.5- to 2-fold [19,59]. At the protein level, the application of small molecule inhibitors that specifically target critical proteins within the NHEJ pathway, such as SCR7 (Ligase IV) and NU7441 (DNA-PKcs) [60,61], as well as agonists of essential proteins in the HDR pathway, such as RS-1 (RAD51) [62], and inhibitors or agonists of other related signaling pathways, such as Brefeldin A, L755507, VE-822, and AZD-7762 [63,64], have been demonstrated to significantly enhance the capacity of HDR in both cells and embryos. Moreover, the overexpression of crucial HDR-promoting proteins like BCL-XL [65], MRE11 [66], CtIP [67], 53BP1 [68], and RAD52 [69], whether administered individually or fusion with the Cas9 protein, markedly improves the HDR efficiency. In addition, modulation of the cell cycle via the administration of small molecules, including Aphidicolin, Nocodazole, and ABT, can facilitate the synchronization of cells to the S and G2/M phases [70,71], during which HDR is most active. Additionally, the fusion of Cas9 with Geminin, a substrate of the E3 ubiquitin ligase complex APC/Cdh1, results in elevated levels of Cas9 protein expression during the S/G2 phase, while expression is diminished in the G1 phase [72]. This cell cycle control strategy can also significantly enhance the exogenous fragment knock-in.

Fetal skin fibroblasts, which serve as the primary donor cells for SCNT in large animals, present significant challenges in enhancing the efficiency of gene knock-in. In a previous study [13], we investigated the effects of several chemical inhibitors targeting the NHEJ pathway—specifically DNA-PKcs inhibitors (PIK-75, SF-2523, and CC-115) and Ligase IV inhibitor (SCR7)—on CRISPR/Cas9-mediated gene knock-in efficiency in SFFs, using the MSTN-T2A-EGFP knock-in system. We found that treatment with PIK-75, SF-2523, and CC-115 did not significantly enhance knock-in efficiency. Although SCR7 showed some improvement, the increase was modest, with efficiency rising from 3.29% ± 0.11% to 3.78% ± 0.25% (*n* = 3, *p* < 0.05). In addition, we identified three small molecules, RITA, Nutlin3, and CTX1, inhibitors of p53-MDM2 interaction, that caused activation of the p53 pathway, resulting in distinct G2/M cell cycle arrest in response to DNA damage and improved the efficiency of exogenous DNA fragment (1730 bp) knock-in from 1.74% to 7.45% at the MSTN locus. Nevertheless, considering the safety requirements for downstream applications involving genome edited animals, it is imperative to employ marker-free gene editing approaches. Additionally, interventions such as small molecule treatments or cell cycle synchronization may perturb normal fibroblast signaling pathways. Inspired by the aforementioned strategy, increasing the local concentration of dsDNA or ssODN donor templates at the site of DSBs induced by the CRISPR system can enhance gene knock-in efficiency, we fused the DNA binding domain of Gal4 and LexA proteins to the C- or N-terminal of the SpCas9 protein, respectively. Additionally, The UAS or op sequence, which can be specifically recognized and bound by Gal4-BD or LexA-BD, respectively, were incorporated at the 5′ or 3′ end of the donor template. It was found that the fusion of Gal4-BD to the N-terminal of the SpCas9 protein (with a linker of 32 aa: SGGS×2-XTEN-SGGS×2) and the incorporation of the UAS at the 3′ end of the donor template led to a notable enhancement in the efficiency of the site-specific integration of exogenous gene, achieving an approximate 3.15-fold increase (from 5.30% (8/151) to 16.67% (32/192), *p* = 3.63 × 10^−3^) without drug selection or fluorescence enrichment. The 32 amino acids form a relatively long linker peptide that, when fully extended, can exceed 10 nanometers in length. This provides ample physical separation between Cas9 and the Gal4-BD protein, significantly reducing steric hindrance. SGGS is a classic, highly flexible linker peptide sequence. The introduction of the XTEN structural element imparts a degree of rigidity, giving the linker a “rod-like” characteristic. This design creates a “spherical-conical activity space”, allowing Gal4-BD substantial freedom of movement and multiple possible orientations. Consequently, this configuration may increase the likelihood of Gal4-BD locating and binding its target DNA site by avoiding confinement to a single unfavorable conformation while also preventing frequent ineffective collisions caused by excessive flexibility. In comparison to the above-mentioned studies, our approach presents several advantages. Specifically, our homologous repair template does not necessitate complex biotin modifications and is composed of dsDNA, which is more suitable for long fragment knock-in applications than ssODN. Moreover, unlike the Cas9/*Sleeping Beauty* system that requires binding to a relatively long 292 bp sequence, our system targets a substantially shorter DNA sequence of only 17 bp. These features enable the preparation of the homologous repair template through simple PCR amplification. Consequently, we successfully developed a novel CRISPR/Cas9-Gal4-mediated gene knock-in system, providing a comparatively efficient and user-friendly strategy for gene knock-in in sheep.

It should be noted that our study has certain limitations. First, when comparing the knockout efficiency of the three Cas systems in SFFs, the same promoter should be used to avoid potential confounding effects caused by differences in promoter activity. Second, CRISPR/Cas9-Gal4 has only been tested at the cellular level in sheep and has not yet been validated in vivo or in embryonic systems. The DNA sequence encoding the Cas9 protein (1368 aa) is 4107 bp in length, while the DNA sequence encoding the Gal4-BD/32 aa/Cas9 fusion protein (147 + 32 + 1367 = 1546 aa) is 4641 bp long. The Gal4-BD/32 aa/Cas9 fusion protein is not significantly larger than the Cas9 protein alone. For embryonic applications, mRNA encoding the Gal4-BD/32 aa/Cas9 fusion protein can be synthesized via in vitro transcription, or the fusion protein itself can be purified using an affinity tag. Delivery to embryos can be achieved through methods such as pronuclear microinjection or electroporation. For in vivo applications, given the need to include additional elements such as the promoter and polyadenylation (polyA) signal—which exceed the packaging capacity of adeno-associated viruses (AAV)—construction of a lentiviral vector may be considered. For applications with stringent biosafety requirements, encapsulating mRNA and donor templates within lipid nanoparticles (LNPs) can provide safe and efficient nucleic acid delivery. Third, the universal applicability of the CRISPR/Cas9-Gal4 system remains to be evaluated. Comprehensive validation across multiple target loci in diverse cell types from humans, mice, goats, and other species is necessary. Fourth, although the CRISPR/Cas9-Gal4 system offers advantages such as ease of use and greater suitability for large-fragment gene knock-in compared to other donor-template tethering systems, no comparative studies have been conducted to determine which system achieves higher gene knock-in efficiency under identical conditions. These limitations are worth further investigating to fully elucidate the capabilities of this genome editing platform in future studies.

## 5. Conclusions

In summary, this study compared the dsDNA cleavage capacity of three representative and commonly used Cas nucleases: SpCas9, AsCas12a, and CasMINI, and identified that SpCas9 exhibited the highest gene knockout efficiency at the cellular level in sheep. Additionally, fusing the Gal4-BD peptide to the N-terminal of the SpCas9 protein through the SGGS×2-XTEN-SGGS×2 flexible linker, along with using a donor template that contains the 17 bp UAS (5′-CGGAAAGCTTCCTTCCG-3′) at the 3′ end, could significantly increase the gene knock-in efficiency by 2.43- (from 5.10% (5/98) to 12.37% (12/97), *p* = 0.13) to 3.15-fold (from 5.30% (8/151) to 16.67% (32/192), *p* = 3.63 × 10^−3^) in SFFs, providing a comparatively efficient and user-friendly method to promote CRISPR-mediated gene knock-in in sheep.

## Data Availability

All data supporting the findings of this study are available in the article and in the Supplementary Data Files. The plasmids and other key resources (such as wild type SFFs, gene knock-in SFFs, etc.) used in this study are available from the corresponding author (Yefeng Qiu, qiuyefeng2001@163.com) upon request. All raw FACS files used in this study have been deposited on Figshare and are available at https://doi.org/10.6084/m9.figshare.30121954.

## Supplementary Materials

The following supporting information can be downloaded at: https://www.mdpi.com/article/10.3390/cimb47110899/s1.
