# Supplementary material for: Improving Gene Knock-In Efficiencies in Sheep Primary Cells Using a CRISPR/Cas9-Gal4 System
Source: Curr Issues Mol Biol. 2025 Oct 29;47(11):899. doi: 10.3390/cimb47110899 (PMC12651790; doi:10.3390/cimb47110899)
Supplement: Supplementary file 1 [file cimb-47-00899-s001.zip › cimb-3920129-supplementary.pdf]

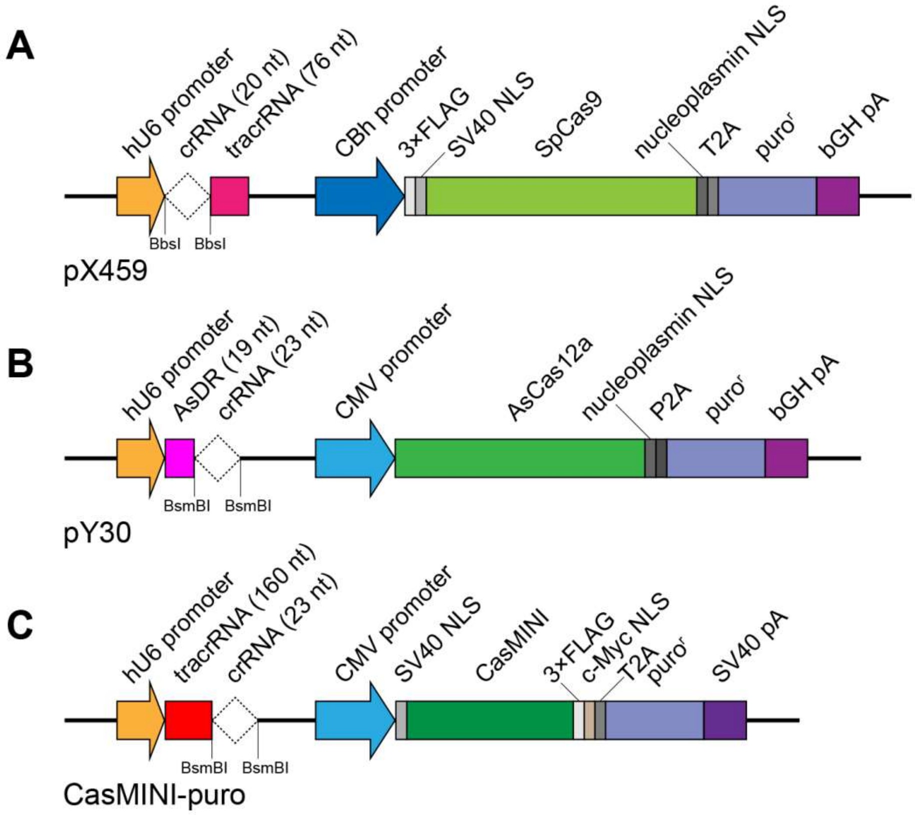

Figure S1. Schematic structures of the three gene knockout plasmids.

In each plasmid, the sgRNA or crRNA (left half) was co-expressed with the Cas protein (right half), and all plasmids additionally contained the puromycin selection gene.

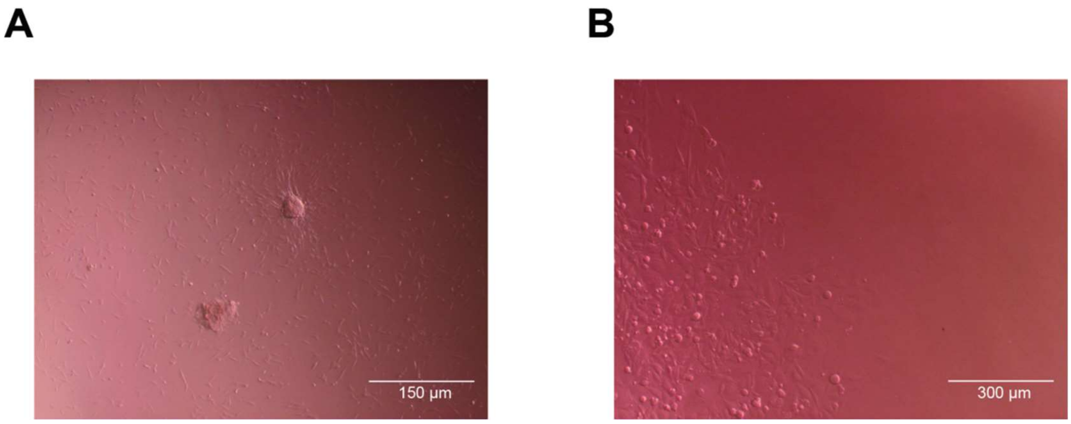

Figure S2. Observing the growth of sheep fibroblasts under the microscope.

(**A**) Microscopic image of isolated sheep fibroblasts. (**B**) Growth status of monoclonal cells on day eight.

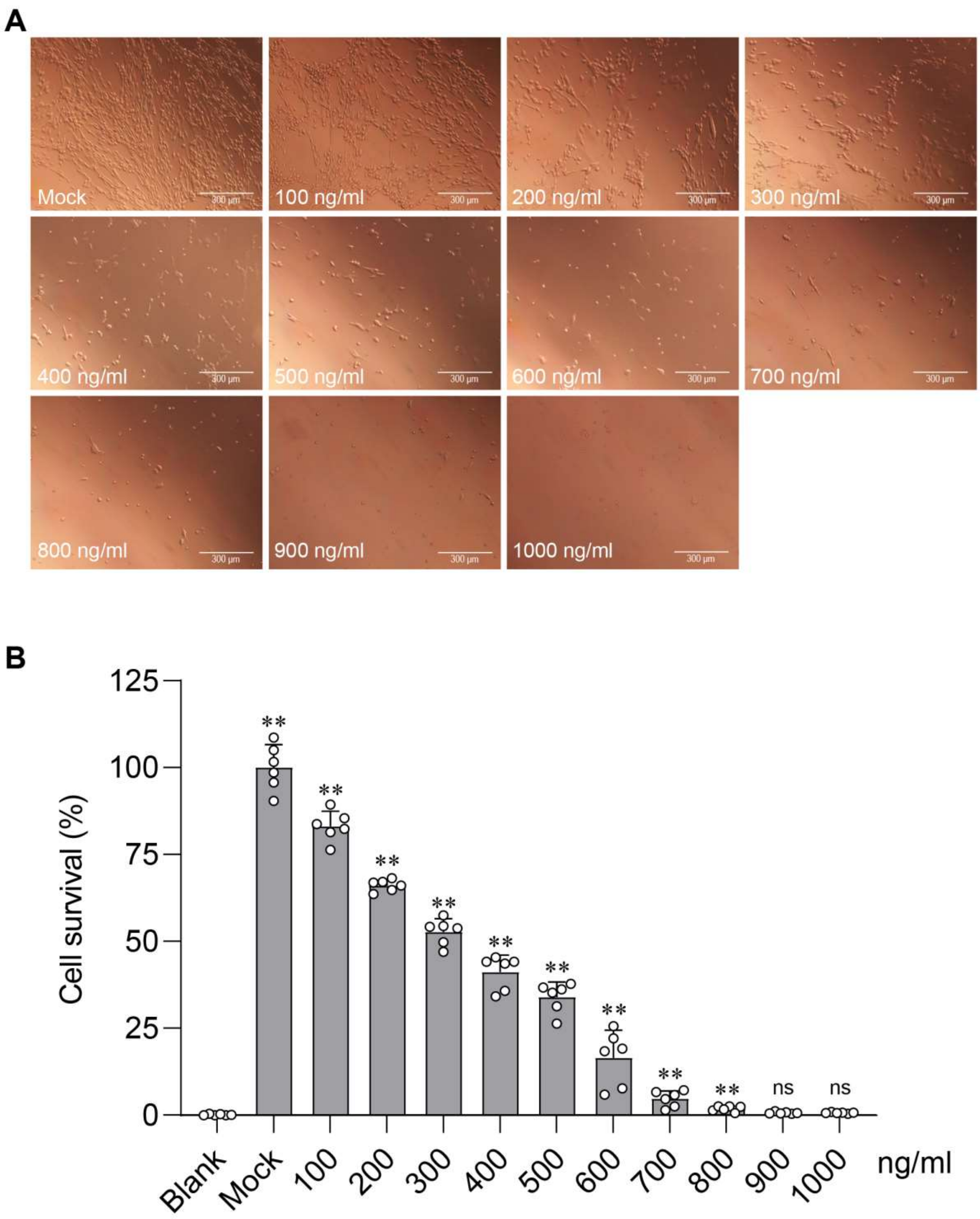

Figure S3. Determination of the minimum lethal dose of puromycin in SFFs.

(**A**) Microscopic observation of cell viability of SFFs following treatment with varying concentrations of puromycin. (**B**) Determination of the cell viability of SFFs treated with different concentrations of puromycin by MTT. Cells without puromycin treatment were used as the control. $n = 6$ biological replicates. Error bars represent SD. Welch's ANOVA was used to assess the significance: ns indicates no significant difference, ** indicates $p < 0.01$.

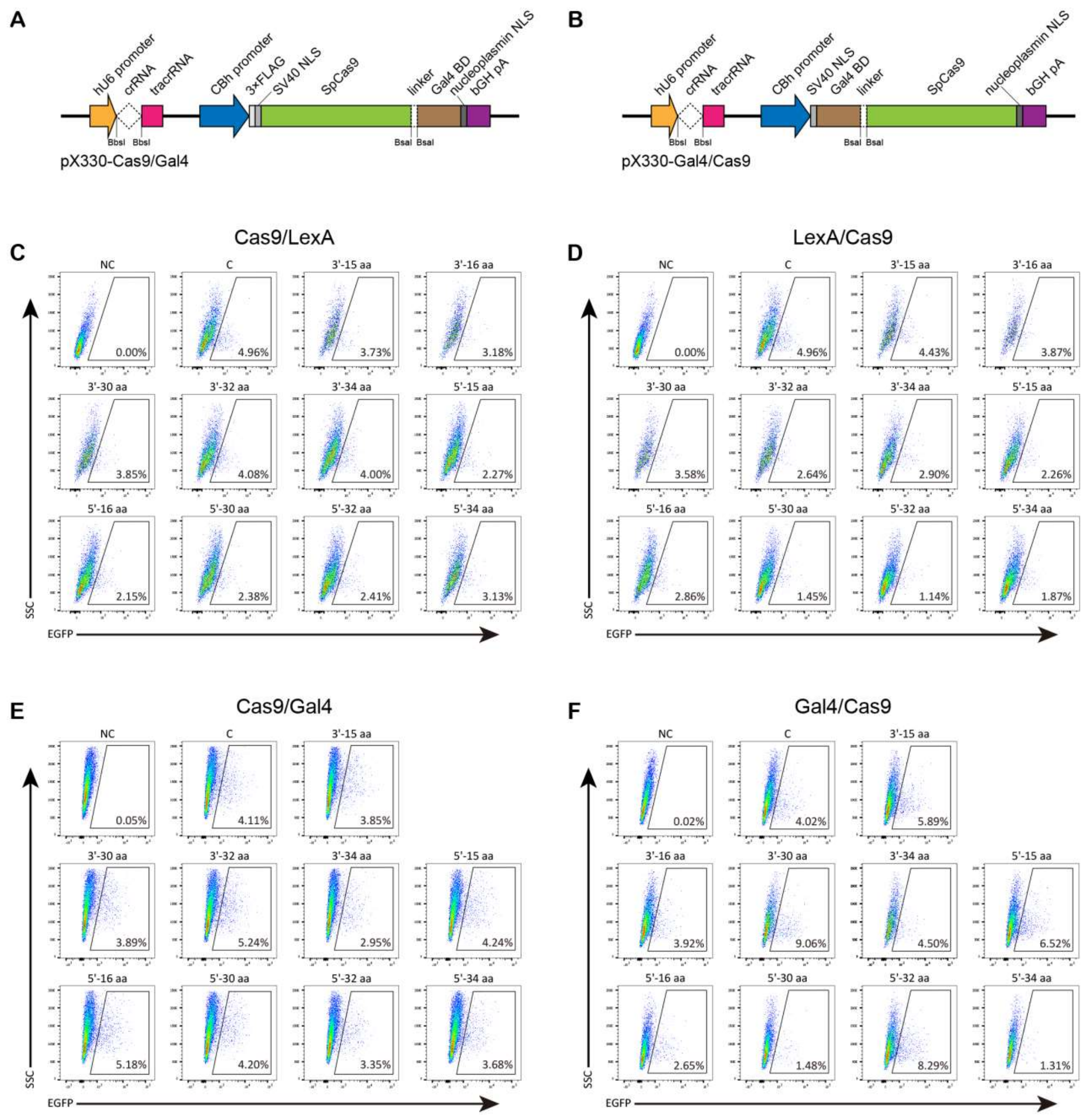

Figure S4. Effect of fusing LexA-BD or Gal4-BD peptide to the C- or N-terminal of Cas9 protein on the efficiency of gene knock-in in SFFs.

(**A**, **B**) Schematic structures of the pX330-Cas9/Gal4 and pX330-Gal4/Cas9 plasmids. Gal4-BD peptide was fused to the C- or N-terminal of the Cas9 protein by five different flexible linkers. (**C**, **D**) Representative FACS results for the negative control, control, Cas9/LexA, and LexA/Cas9 groups are presented. (**E**, **F**) Representative FACS results for the negative control, control, Cas9/Gal4, and Gal4/Cas9 groups are presented.

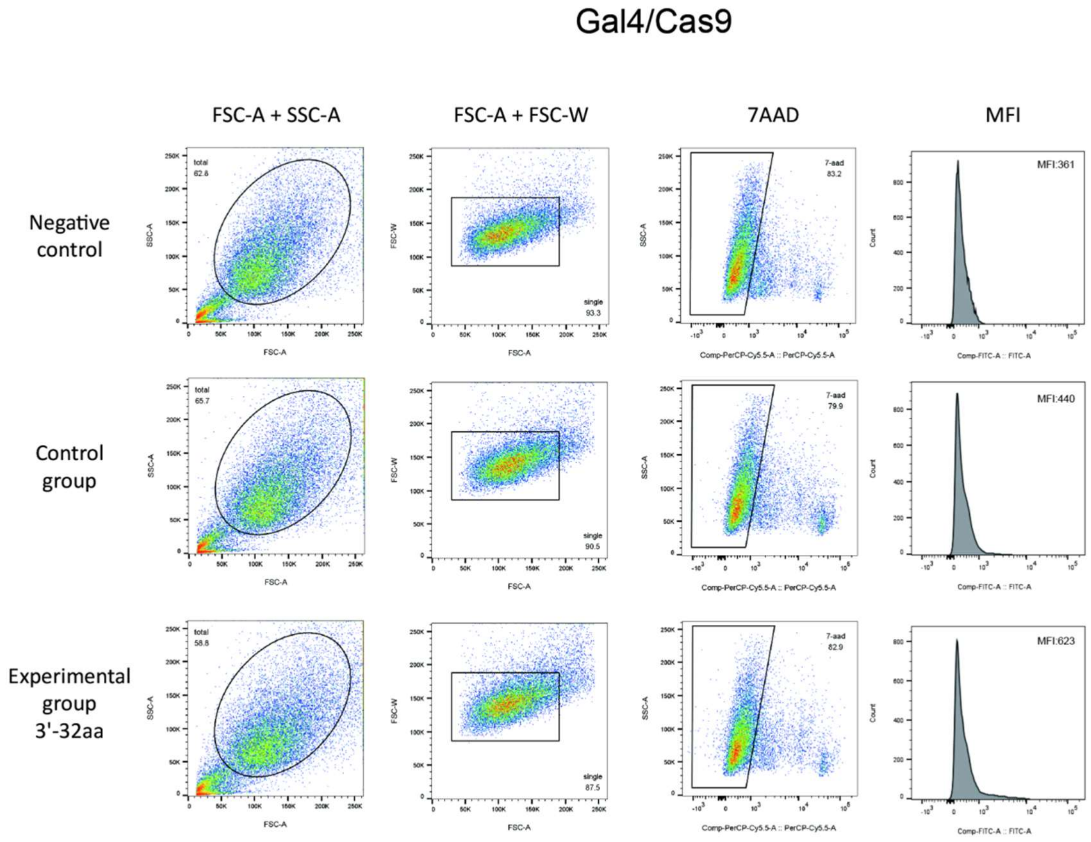

Figure S5. Representative FACS gating plots (FSC/SSC, single-cell, 7-AAD (live cells), and fluorescence intensity histogram) for each group in the Gal4/Cas9-mediated gene knock-in assay.

A

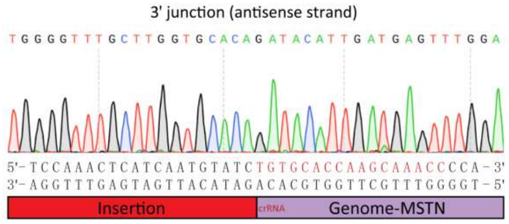

B

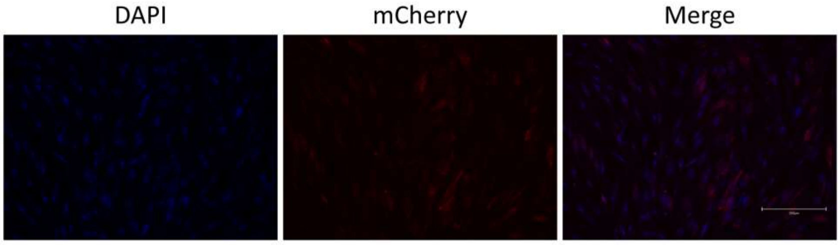

C

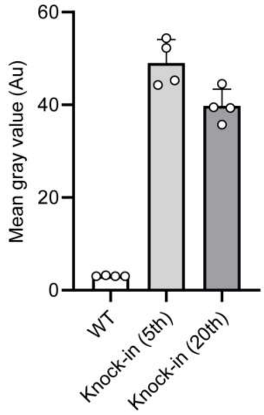

D

Binding sites		sgRNA guiding sequence and base position																							Indel
sgRNA	MSTN location	1	2	3	4	5	6	7	8	9	10	11	12	13	14	15	16	17	18	19	20	P	A	M	
on	chr2-119290900-119290922	G	G	T	T	T	G	C	T	T	G	G	T	G	C	A	C	A	A	G	A	T	G	G	
off-1	chr3-156001406-156001428	G	G	T	T	T	A	C	T	T	G	G	T	G	C	C	C	A	T	G	A	C	G	G	no
off-2	chr3-74335157-74335179	G	G	T	T	T	G	C	T	T	T	G	T	G	C	A	A	A	A	G	A	G	G	G	no
off-3	chr12-65148801 to 65148823	G	G	T	T	T	G	G	T	T	G	A	T	G	C	A	C	A	A	A	A	A	G	G	no
off-4	chr13-18170749-18170771	G	G	T	T	T	G	T	T	T	G	G	G	G	A	A	C	A	A	G	A	G	G	G	no
off-5	Chr24-39525417-39525439	G	G	T	T	T	A	G	T	T	G	G	T	G	C	A	C	A	C	G	A	A	G	G	no

.

Figure S6. Sequencing, fluorescent expression analysis, average fluorescence intensity measurement, and off-target detection results for mCherry gene knock-in cell lines.

(**A**) Sanger sequencing of the 3'-junction region (CMV-mCherry-pA) upon correct targeting at the *MSTN* locus. (**B**) Fluorescent expression of the mCherry gene in the 20th-generation monoclonal sheep fibroblast cell line. (**C**) Average fluorescence intensity of the mCherry gene in monoclonal sheep fibroblast cell lines at the 5th and 20th generations. (**D**) Off-target analysis of sites similar to MSTN sgRNA2 sequences.

A

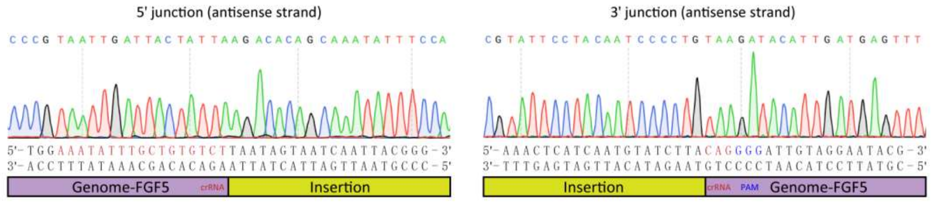

B

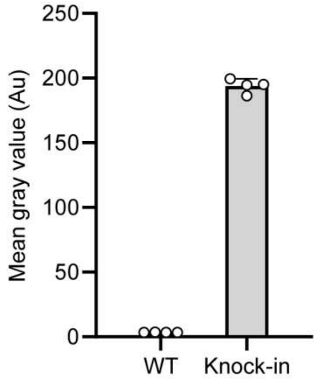

C

Binding sites		sgRNA guiding sequence and base position																							Indel
sgRNA	FGF5 location	1	2	3	4	5	6	7	8	9	10	11	12	13	14	15	16	17	18	19	20	P	A	M	
on	Chr6- 95677501- 95677520	A	A	A	T	A	T	T	T	G	C	T	G	T	G	T	C	T	C	A	G	N	G	G	
off-1	Chr1-234182001-234182023	A	A	A	T	A	T	T	T	G	C	T	C	T	G	T	C	T	C	A	A	T	G	G	no
off-2	Chr2-189379244-189379266	A	A	A	T	G	T	A	T	G	C	T	G	A	G	T	C	T	C	A	G	T	G	G	no
off-3	Chr10-46175183- 46175205	T	A	A	C	A	T	T	T	G	C	T	G	T	G	T	C	T	C	A	T	A	G	G	no
off-4	Chr14-59873030-59873052	A	A	A	T	A	A	T	T	G	C	T	C	T	G	T	G	T	C	A	G	G	G	G	no
off-5	Chr15-15049934-15049956	A	A	A	G	T	T	T	T	G	C	T	G	T	G	T	C	T	G	A	G	G	G	G	no

Figure S7. Sequencing, average fluorescence intensity measurement, and off-target detection results for SV40 LT gene knock-in cell lines.

(**A**) Sanger sequencing of the 5'- and 3'-junction regions (CMV-SV40LT-pA) upon correct targeting at the *FGF5* locus. (**B**) Average fluorescence intensity of the SV40 LT gene in monoclonal sheep fibroblast cell lines at the 5th generation. (**C**) Off-target analysis of sites similar to FGF5 sgRNA2 sequences.

Table S1. Primers used in this study

Primer/Target name	Direction	Sequence (5′ to 3′)	Assays
Cas9-MSTN-1	F	CACCGCTATTTATGCTGCTTGTTGC	Construction of the pX459 pX330, and pX330-Gal4/32 aa/Cas9 gene konck out plasmids
	R	AAACGCAACAAGCAGCATAAATAGC	
Cas9-MSTN-2	F	CACCGGGTTTGCTTGGTGCACAAGA	
	R	AAACTCTTGTGCACCAAGCAAACCC	
Cas9-FGF5-1	F	CACCGCATCCGGGTAGATCTGCAGA	
	R	AAACTCTGCAGATCTACCCGGATGC	
Cas9-FGF5-F2	F	CACCGAAATATTTGCTGTGTCTCAG	
	R	AAACCTGAGACACAGCAAATATTTC	
Cas9-MSTN	F	CACCGGACATCTTTGTAGGAGTACAGCAA	Construction of the pX330-LexA/linker/Cas9, pX330-Cas9/linker/LexA, pX330-Gal4/linker/Cas9, and pX330-Cas9/linker/Gal4 plasmids with different linker lengths
	R	AAACTTGCTGTACTCCTACAAAGATGTCC	
Cas12a-MSTN-1	F	AGATCCTATTTATGCTGCTTGTTGCTGTT	Construction of the pY30 gene konck out plasmids
	R	AAAAAACAGCAACAAGCAGCATAAATAGG	
Cas12a-MSTN-2	F	AGATGGGTTTGCTTGGTGCACAAGATGTT	
	R	AAAAAACATCTTGTGCACCAAGCAAACCC	
Cas12a-FGF5-1	F	AGATCCATCCGGGTAGATCTGCAGATGTT	
	R	AAAAAACATCTGCAGATCTACCCGGATGG	
Cas12a-FGF5-2	F	AGATGAAATATTTGCTGTGTCTCAGGGTT	
	R	AAAAAACCCTGAGACACAGCAAATATTTC	

CasMINI-MSTN-1	F	GCAACCCTATTTATGCTGCTTGTTGCTG	Construction of the CasMINI-purogene konck out plasmids
	R	AAAACAGCAACAAGCAGCATAAATAGGG	
CasMINI-MSTN-2	F	GCAACGGGTTTGCTTGGTGCACAAGATG	
	R	AAAACATCTTGTGCACCAAGCAAACCCG	
CasMINI-FGF5-1	F	GCAACCCATCCGGGTAGATCTGCAGATG	
	R	AAAACATCTGCAGATCTACCCGGATGGG	
CasMINI-FGF5-2	F	GCAACGAAATATTTGCTGTGTCTCAGGG	
	R	AAAACCCTGAGACACAGCAAATATTTCG	
MSTN target-1	F	GGCAGGCATTAACGTTTGGCTT	Detection of the indel frequency
	R	CTGATCAATCAGTTCCCGGAGT	
MSTN target-2	F	GAAGCTTTTGGATGGGATTGGAT	
	R	ACTCTAGGCTTATAGCCCGTGGT	
FGF5 target-1	F	CAGTAGCACCGTGTCTTCCTCTT	
	R	CAAGCTCCGGAAGGTGCGCTTAC	
FGF5 target-2	F	AAACTCACTGTAATAAAGAATGG	
	R	TAAAGTGCTCTGGGCAAATTGTC	
MSTN/sgRNA-1000 HA	F	TCTCAAATTCATGAAAAGATTGG	Generation of the donor templates
	R	AATCTTTTTATACAATATTGATAG	
LexA/op-MSTN/sgRNA-1000 HA	F	TACTGTATGATCATACAGTATCTCAAATTCATGAAAAGATTGG	
	R	TACTGTATGATCATACAGTAAATCTTTTTATACAATATTGATAG	
Gal4/UAS-MSTN/sgRNA-1000 HA	F	CGGAAAGCTTCCTTCCGTCTCAAATTCATGAAAAGATTGG	
	R	CGGAAAGCTTCCTTCCGAATCTTTTTATACAATATTGATAG	
MSTN/sgRNA2-1000 HA	F	GATGTATTCCTCAGAATTTTCCA	
	R	TTATTTCATCCTAAAAGCTGCAG	
Gal4/UAS-MSTN/sgRNA2-1000 HA	R	CGGAAAGCTTCCTTCCGTTATTTCATCCTAAAAGCTGCAG	

FGF5/sgRNA2-2000 HA	F	TTTGTATCTGTCATTCTACACAT	
	R	CTTTTAGCCTGCCAAGATGAGAA	
Gal4/UAS-FGF5/sgRNA2-2000 HA	R	CGGAAAGCTTCCTTCCGCTTTTAGCCTGCCAAGATGAGAA	
#1	F	CCCTGAACCTGAAACATAAAATG	Genotyping
	R	TCACGAACCCATAAGTGAATGCT	
#2	F	GTCTTGACCTCTTAAATGTTAAC	
	R	CCGTAAGTTATGTAACGCGGAAC	
#3	F	CACTGCATTCTAGTTGTGGTTTG	
	R	AGACAGTGGGCTTGGACAATTGG	

Table S2. Plasmids used in this study

Primer/Target name	Description	Assays
pX330	sgRNA and Cas9 co-expression (Addgene plasmid 42230)	T2A-EGFP, mCherry, and SV40 LT gene knock-in (Figure 2, Figure 3, Figure 4)
pX459	sgRNA, Cas9, and puromycin co-expression (Addgene plasmid 48139)	MSTN and FGF5 gene knock out (Figure 1)
pY30	crRNA, Cas12a, and puromycin co-expression (Addgene plasmid 84745)	
CasMINI-Puro	sgRNA, CasMINI, and puromycin co-expression	
pEGFP-N1	Commercial plasmid (Clontech)	Construction of the CasMINI-Puro plasmid
pX330-LexA/Cas9	Modifications conducted using the pX330 plasmid	LexA-BD fused to the N-terminal region of the Cas9 protein (no linker)
pX330-Cas9/LexA		LexA-BD fused to the C-terminal region of the Cas9 protein (no linker)
pX330-Gal4/Cas9		Gal4-BD fused to the N-terminal region of the Cas9 protein (no linker)
pX330-Cas9/Gal4		Gal4-BD fused to the C-terminal region of the Cas9 protein (no linker)
pX330-LexA/linker/Cas9	Five flexible linkers of varying lengths were connected to each plasmid.	Screen for the linker with the highest knock-in efficiency (Figure 2)
pX330-Cas9/linker/LexA		
pX330-Gal4/linker/Cas9		
pX330-Cas9/linker/Gal4		
pX330-Gal4/32 aa/Cas9	Fusion of the Gal4-BD to the N-terminal of the Cas9 protein through the 32 amino acid linker peptide (SGGS×2-XTEN-SGGS×2)	mCherry, and SV40 LT gene knock-in (Figure 2)
MSTN/mCherry donor	Generation of the CMV-mCherry-pA with 1000 bp HA repair template	mCherry gene knock-in (Figure 3)
pmCherry-N1	Commercial plasmid (Clontech)	Construction of the MSTN/mCherry donor plasmid and FGF5/SV40 LT donor plasmid

FGF5/SV40 LT donor	Generation of the CMV-SV40 LT-pA with 2000 bp HA repair template	SV40 LT gene knock-in (Figure 4)
pBABE-puro SV40 LT	Containing the SV40 LT gene expression construct (Addgene plasmid 13970）	Construction of the FGF5/SV40 LT donor plasmid

Table S3. Summary of gene knock-in efficiency in sheep at MSTN and FGF5 loci using the three CRISPR systems: Cas9, LexA/Cas9, and Gal4/Cas9.

	T2A-EGFP knock-in efficiency (MSTN)	mCherry knock-in efficiency (MSTN)	SV40 LT knock-in efficiency (FGF5)
Cas9 vs LexA/Cas9	4.58% ± 0.45%<br>vs<br>4.34% ± 0.23%	/	/
Cas9 vs Gal4/Cas9	4.05% ± 0.19%<br>vs<br>10.50% ± 0.17%	5.10%<br>vs<br>12.27%	5.30%<br>vs<br>16.67%
